# School-based physical activity and health-related fitness in Mediterranean students: findings from the DELICIOUS project

**DOI:** 10.3389/fpubh.2025.1603043

**Published:** 2025-06-03

**Authors:** Mohamed Aly, Achraf Ammar, Khaled Trabelsi, Liwa Masmoudi, Noha El-Gyar, Amira M. Shalaby, Osama Abdelkarim

**Affiliations:** ^1^Faculty of Sport Sciences, Assiut University, Assiut, Egypt; ^2^Department of Training and Movement Science, Institute of Sport Science, Johannes Gutenberg University Mainz, Mainz, Germany; ^3^High Institute of Sport and Physical Education of Sfax, University of Sfax, Sfax, Tunisia; ^4^Research Laboratory, Molecular Bases of Human Pathology, Faculty of Medicine of Sfax, University of Sfax, Sfax, Tunisia; ^5^Research Laboratory Education, Motricity, Sport and Health, EM2S, High Institute of Sport and Physical Education of Sfax, University of Sfax, Sfax, Tunisia; ^6^Department of Movement Sciences and Sports Training, School of Sport Science, The University of Jordan, Amman, Jordan; ^7^Faculty of Medicine, Department of Pediatrics, Assiut University, Assiut, Egypt

**Keywords:** physical fitness, exercise intervention, Mediterranean region, cross-cultural study, youth health promotion, early adolescents, children, anthropometrics

## Abstract

**Background:**

Physical inactivity among children is a growing public health concern, particularly in Mediterranean countries, where lifestyle changes have contributed to declining physical fitness levels. Structured school-based interventions have shown promise in improving children’s health-related physical fitness (HRF), but cross-cultural differences in intervention effectiveness remain understudied. This study, conducted within the DELICIOUS project, evaluates the impact of a standardized physical activity (PA) intervention on HRF components among children (8–10 years) and early adolescents (11–14 years) from five Mediterranean countries: Egypt, Italy, Lebanon, Portugal, and Spain.

**Methods:**

A total of 937 participants aged 8–14 years took part in a six-month school-based PA program designed to enhance speed, agility, muscular strength, cardiovascular endurance, and coordination. Physical fitness was assessed using the International Physical Performance Test Profile (IPPTP) before and after the intervention. A three-way repeated measures ANOVA (Time × Age × Country) assessed intervention effects on anthropometric and fitness variables, while a two-way ANOVA (Age × Country) examined the percentage changes in these HRF across age groups and countries.

**Results:**

Significant improvements were observed across multiple health-related fitness components, particularly in speed, lower-body power, coordination, muscular endurance, and cardiovascular endurance. Early adolescents (11–14 years) generally showed greater gains than younger children (8–10 years). The magnitude of improvement in fitness outcomes varied by country. Lebanon and Portugal recorded the most substantial gains in sprint, strength, and endurance. Spain and Italy showed relatively smaller improvements, especially among younger participants, whereas Egyptian students demonstrated notable gains in sprint performance and endurance, particularly among early adolescents, along with the most significant BMI reduction observed in the study. BMI remained stable across participants, suggesting that fitness improvements were achieved alongside healthy growth, without adverse changes in body composition.

**Conclusion:**

A structured PA intervention can effectively improve HRF in children across Mediterranean countries, though outcomes vary by age and cultural context. These findings highlight the need for tailored, school-based PA programs that consider baseline fitness levels and regional factors. Implementing such interventions could play a crucial role in addressing physical inactivity and fostering long-term health benefits in children.

## 1 Introduction

Physical activity (PA) during childhood is a cornerstone of healthy development, offering physical, mental, and cognitive benefits (1, 2). Regular engagement in PA in children and adolescents improves cardiorespiratory fitness, muscular strength, bone health, coordination, and metabolic function. It also contributes to cognitive outcomes such as memory, attention, and academic performance (3–5). Conversely, insufficient activity increases the risk of obesity and related noncommunicable diseases (6) even from an early age (7, 8). The World Health Organization (WHO) reports that approximately 80% of adolescents globally fail to meet the recommended 60 min of moderate-to-vigorous PA per day (9). This inactivity crisis has contributed to a documented decline in youth physical fitness over the past few decades. For example, longitudinal analyses have revealed substantial drops in children’s cardiorespiratory endurance (a key indicator of aerobic fitness) since the 1980s, reflecting a worrying erosion in health-related fitness (HRF) levels across many countries (10–12). Recent evidence suggests that this inactivity crisis has been further exacerbated by the COVID-19 pandemic, which led to widespread school closures, increased sedentary behavior, and reduced access to structured PA for children and adolescents (13–15). These trends underscore the urgent need for effective interventions to promote PA and improve health-related fitness in young populations.

HRF in youth typically encompasses several fundamental components: muscular strength (including power and endurance), cardiorespiratory endurance, speed, agility, coordination, flexibility, and healthy body composition (16). Higher fitness levels during childhood are associated with healthier body weight, improved cardiovascular profiles, and better mental health outcomes in later life (17). Additionally, components such as speed and agility reflect motor skill development (18), while strength and endurance support functional independence (19, 20). Coordination enables complex movements and overall physical proficiency. Developing these skills early can influence lifelong adherence to active lifestyles and reduce the risk of chronic diseases (21, 22). To evaluate these fitness components in children, various standardized assessment protocols have been developed and validated in the pediatric population. These tools aim to provide comprehensive, age-appropriate measures of fitness across domains such as strength, speed, endurance, and coordination. One notable example is the ASSO Project, which proposed a Fitness Index model specifically designed for Italian adolescents living in Southern Italy and provided valuable normative data within that context (23). Such tools emphasize the importance of culturally adapted and validated assessments and highlight the value of comparing data across different populations using standardized instruments. These considerations reinforce the need for employing robust, cross-culturally applicable tools—such as the International Physical Performance Test Profile (IPPTP)—in multi-national studies.

Despite the known benefits of PA, many children, particularly in certain regions, are not achieving adequate fitness. In Mediterranean countries, historically associated with active lifestyles and healthy diets, recent data show high rates of childhood overweight and obesity (24). Sedentary behaviors and dietary shifts have led to declining activity levels and reduced physical fitness (25–27). For example, surveillance data show that about one-third of children aged 6 to 9 in Southern Europe are overweight, with prevalence particularly high in countries like Italy, Spain, Greece, and Cyprus (28). These trends present important public health challenges.

Structured interventions, particularly in school settings, have emerged as key strategies to counteract these negative trends. Numerous studies have shown that school-based PA programs can enhance various components of fitness, such as aerobic capacity, muscular strength, and motor coordination (29–32). Systematic reviews support the effectiveness of frequent physical education sessions in improving children’s health-related fitness (33). Multi-component programs that combine aerobic exercise, strength training, and skill-based activities have also proven effective, even within relatively short periods (34, 35). However, intervention outcomes may vary depending on baseline fitness levels and lifestyle factors. Children with lower initial fitness often show greater improvements, while those who are already active may experience smaller gains. Cultural and environmental factors also influence program effectiveness due to differences in school curricula, access to extracurricular sports, and general attitudes toward PA (36, 37). These findings suggest the need to test interventions in diverse settings, particularly in culturally heterogeneous regions like the Mediterranean.

This study is conducted within the framework of the DELICIOUS project—a multinational initiative aimed at promoting healthy Mediterranean diet and lifestyle (including regular PA) among children in Mediterranean countries (38). DELICIOUS (an acronym for “Diet and Eating habits, Lifestyle Interventions for Children In secOndary schools and primary schools to Upraise Mediterranean Sustainable food habits,” a hypothetical expansion) is a 3-year project supported by the EU PRIMA program, involving five countries: Egypt, Italy, Lebanon, Portugal, and Spain (39). A key objective of DELICIOUS is to counteract the decline in Mediterranean children’s fitness and dietary habits by implementing school-based interventions that encourage more active lifestyles and better nutrition.

In the present study, we present a comparative analysis of the effect of the DELICIOUS PA intervention on health-related fitness components in children from five Mediterranean countries. To our knowledge, this is one of the first multi-country studies to evaluate a unified PA program in a Mediterranean context, allowing cross-cultural comparisons of intervention effectiveness. The specific objectives of the study are: (1) to determine the extent to which the intervention improves each of the targeted fitness components (speed, agility, muscular strength, cardiovascular endurance, and coordination) in children; and (2) to compare the magnitude of these fitness changes across the five countries, and between two age groups (children aged 8–10 vs. early adolescents aged 11–14), enabling more tailored practical recommendations. We hypothesize that all participating countries will show improvements in most fitness measures after the intervention, but the degree of improvement may vary, with potentially larger gains in settings with lower baseline fitness or less prior exposure to organized PA. By addressing these aims, the study seeks to inform how school-based PA interventions can be adapted for diverse cultural settings, with implications for public health. Its findings would help guide strategies to improve youth fitness and reduce inactivity, ultimately supporting healthier adult populations and global health goals.

## 2 Materials and methods

### 2.1 Participants

The study sample was drawn from partner countries within the DELICIOUS consortium: Egypt, Italy, Lebanon, Portugal, and Spain. Primary goals and objectives of the DELICIOUS project, a comprehensive descriptive and intervention study aimed at promoting the Mediterranean diet and an active lifestyle among school children and adolescents, have been detailed elsewhere (38, 40). A formal sample size calculation was not performed prior to recruitment. Instead, a pragmatic approach was adopted, aiming to enroll the maximum number of eligible participants across the five participating Mediterranean countries. Schools were selected based on willingness and logistical feasibility, and the majority of eligible children within participating schools were invited to take part in the study. Thus, a convenience sampling strategy was employed to balance scientific rigor with practical constraints related to project timelines, resources, and field implementation across multiple countries. Accordingly, a total of 937 children and early adolescents aged 8 to 14 years were recruited from participating schools in urban areas across five Mediterranean cities: Assiut (Egypt), Lisbon (Portugal), Beirut (Lebanon), Cordoba (Spain), and Giugliano in Campania (Italy). To account for potential age-related differences in fitness levels, participants were stratified into two age groups: 8–10 years (children) and 11–14 years (early adolescents). Written informed consent was obtained from parents or legal guardians of all participants prior to the children’s participation. The study adhered to the ethical standards outlined in the Declaration of Helsinki and was approved by the ethics committee of Mondragon University (no. IEB-20230704).

### 2.2 Physical activity intervention program

A standardized, school-based physical activity intervention program was developed within the DELICIOUS project and implemented across all participating Mediterranean countries. Inspired by evidence-based models such as “Build Our Kids’ Success (BOKS)” (41), the program aimed to enhance key components of physical fitness—speed, agility, strength, endurance, and coordination—through structured, age-appropriate, and non-competitive activities that emphasize personal progress and inclusivity, particularly for children who are typically less active. The intervention was delivered over a six-month period and included a comprehensive series of activity plans targeting functional movement skills. Each session followed a standardized structure comprising a warm-up, skill instruction, movement-based exercises, and engaging games designed to reinforce the targeted skills. Sessions were conducted three times per week, with each lasting between 40 and 45 min. Implementing a uniform program across culturally distinct settings allowed for the examination of cross-cultural variability in outcomes and intervention effectiveness.

### 2.3 Measures

#### 2.3.1 International Physical Performance Test Profile

The International Physical Performance Test Profile (IPPTP) is a standardized and validated test battery designed to assess HRF in children and adolescents. It provides a comprehensive evaluation of key fitness components, including speed, agility, strength, endurance, and coordination, making it particularly well-suited for cross-cultural studies (29, 42–45). Developed based on the methodologies of Bös and Mechling (46) and the German Motor Test 6–18 (47), the IPPTP comprises eight test items that encompass the five fundamental dimensions of physical fitness: endurance, strength, speed, coordination, and flexibility. These tests are widely recognized as valid and reliable field-based measures of core physical fitness components in pediatric populations. Prior research, including Bianco et al. (23), demonstrated strong correlations between these tests and other health-related fitness components in adolescents, confirming their suitability for large-scale youth fitness evaluations. For this study, six specific tests from the IPPTP were selected to target the core aspects of physical fitness relevant to children’s HRF. These tests were chosen for their high validity in measuring fitness components across culturally diverse populations and their robustness in cross-cultural applications. For this study, six tests were selected to target core aspects of children’s HRF: the 20 m Dash test assessed speed by recording sprint times over 20 meters; the Sideways Jumping (JSW) test measured agility and coordination through the number of lateral jumps in 15 s; the Push-Up (PU) test evaluated upper body strength and endurance by counting the total push-ups completed in 40 s; the Sit-Up (SU) test assessed core strength through the number of sit-ups performed in 40 s; the Standing Long Jump (SLJ) test measured lower body strength by the distance jumped from a standing position in centimeters; and the 6-Minute Run test assessed cardiovascular endurance by the total distance covered in meters. All tests were conducted under standardized conditions to ensure consistency and reliability across the diverse sample populations. The reproducibility and validity of the IPPTP have been established in previous research (29, 42). For additional details on these test items, readers are referred to the corresponding manuals (44).

#### 2.3.2 Anthropometry

Height and weight were recorded before testing. Height was measured to the nearest 0.1 cm using a portable stadiometer (Seca 213, Seca GmbH & Co. KG, Hamburg, Germany), while weight was measured using a Tanita digital body composition scale (BF-350 Total Body Composition Analyzer, Amsterdam, the Netherlands), following standardized anthropometric protocols. Body mass index (BMI) was then calculated using the formula: BMI = weight (kg)/height (m^2^).

#### 2.3.3 Statistical analysis

All statistical analyses were conducted using SPSS (version 25). The normality of the data was verified using the Shapiro–Wilk test. A three-way repeated measures ANOVA (2 levels for time: pre- and post-intervention × 2 levels for age groups: 8–10 years and 11–14 years × 5 levels for countries) was performed to evaluate the effects of the intervention program on body weight, height, BMI, and various physical fitness variables across different age groups and countries using pre- and post-intervention data. Additionally, a two-way ANOVA (2 levels for age groups × 5 levels for countries) was applied to assess the effects of the intervention program on the same parameters using percentage changes from pre- to post-intervention. Post-hoc differences were investigated using the Bonferroni test to identify pairwise comparisons between groups. Effect size was calculated using partial eta squared (η_p_^2^) to evaluate the magnitude of change. Statistical significance was set *a priori* at *p* < 0.05 for all analyses.

## 3 Results

A total of 354 children (aged 8–10 years) and 583 early adolescents (aged 11–14 years) were included in the final analysis. Participants were distributed as follows: 204 from Egypt (49 children and 155 early adolescents), 200 from Lebanon (92 children and 108 early adolescents), 150 from Italy (111 children and 39 early adolescents), 202 from Spain (32 children and 170 early adolescents), and 181 from Portugal (70 children and 111 early adolescents).

### 3.1 Effects of the intervention program on body weight, height, and BMI across different age groups and countries

Table 1 presents the effects of time (pre- and post-intervention), age, and country of living on weight, height, and BMI. The three-way repeated measures ANOVA revealed significant Time × Age × Country interactions for weight, height, and BMI (*p* = 0.03, *p* < 0.01, and *p* = 0.02, respectively), as well as significant main effects of sex and country on all three variables (*p* < 0.001). A significant main effect of time was observed for weight and height (*p* < 0.001), but not for BMI (*p* = 0.08). Post-hoc analysis showed that weight increased significantly after the intervention in Lebanon and Portugal across both age groups (*p* < 0.05), in Italy only for the 8–10 years age group (*p* < 0.05), and in Spain only for the 11–14 years age group (*p* < 0.05). No significant weight changes were observed in any age group among Egyptian participants (*p* > 0.05). Height significantly increased from pre- to post-intervention in both age groups in most countries (*p* < 0.05), except in Italy, where this difference was significant only for the 8–10 years age group (*p* < 0.05). Participants aged 11–14 years showed significantly higher weight and height values compared to those aged 8–10 years (*p* < 0.05) in most countries, except in Italy. The highest weight and height values across both age groups were recorded in Spain.

**Table 1:** Body weight, height, and BMI at pre- and post-intervention categorized by age-group and countries.

Variables	Age group	Country	Mean/SD		Main effect			Significant interaction
			Pre	Post	Time	Age	Country	
Weight (kg)	8–10 years	Egypt	36.9 ± 9.5	37.4 ± 8.8	*F*_(1, 910)_ = 250.31; *p* < 0.001; η_p_^2^ = 0.22	*F*_(1, 910)_ = 157.64; *p* < 0.001; η_p_^2^ = 0.15	*F*_(4, 910)_ = 15.8; *p* < 0.001; η_p_^2^ = 0.06	Age × Country [*F*_(4, 910)_ = 9.52; *p* < 0.001; η_p_^2^ = 0.04], Time × Country [*F*_(4, 910)_ = 21.15; *p* < 0.001; η_p_^2^ = 0.09] and Time × Age × Country [*F*_(4, 910)_ = 2.74; *p* = 0.028; η_p_^2^ = 0.01]
		Lebanon	33.5 ± 8.2	35.5 ± 9.5^*^				
		Italy	38.8 ± 9.8	40.1 ± 9.7^*^				
		Spain	44.2 ± 10.6 L	44.4 ± 10.2 L				
		Portugal	32.2 ± 6.7IS	33.5 ± 7.1^*^IS				
	11–14 years	Egypt	46 ± 11.8Y	46.5 ± 11.1Y				
		Lebanon	49.6 ± 11.7Y	52.1 ± 11.7^*^YE				
		Italy	41 ± 9.5 L	41.6 ± 8.9 L				
		Spain	53.3 ± 12.5YEI	54.5 ± 12.3^*^YEI				
		Portugal	45 ± 9.7YS	46.5 ± 10.2^*^YLS				
Height (cm)	8–10 years	Egypt	141.3 ± 7.4	143.4 ± 7.4^*^	*F*_(1, 910)_ = 1669.7; *p* < 0.001; η_p_^2^ = 0.65	*F*_(1, 910)_ = 386.37; *p* < 0.001; η_p_^2^ = 0.3	*F*_(4, 910)_ = 28.42; p < 0.001; η_p_^2^ = 0.11	Age × Country [*F*_(4, 910)_ = 18.62; *p* < 0.001; η_p_^2^ = 0.08], Time × Age [*F*_(1, 910)_ = 33.62; *p* < 0.001; η_p_^2^ = 0.04], Time × Country [*F*_(4, 910)_ = 41.4; *p* < 0.001; η_p_^2^ = 0.15] and Time × Age × Country [*F*_(4, 910)_ = 9.13; *p* < 0.001; η_p_^2^ = 0.04]
		Lebanon	137.8 ± 7.8	139.9 ± 8.2^*^				
		Italy	140.3 ± 7.7	141.1 ± 7.6^*^				
		Spain	146.6 ± 7.7 L	148.9 ± 7.5^*^LI				
		Portugal	136.1 ± 7.6S	139 ± 8.2^*^S				
	11–14 years	Egypt	152.8 ± 10.5Y	154.8 ± 10.4^*^Y				
		Lebanon	157.5 ± 8.8YE	159.3 ± 8.7^*^YE				
		Italy	143.6 ± 7.2EL	144 ± 7.1EL				
		Spain	160.9 ± 10.5YEI	163.1 ± 10.4^*^YEI				
		Portugal	155.2 ± 9.1YIS	156.6 ± 9.6^*^YIS				
BMI (kg.m^−2^)	8–10 years	Egypt	18.3 ± 3.8	18.1 ± 3.3	*F*_(1, 910)_ = 3.06; *p* = 0.08; η_p_^2^ = 0	*F*_(1, 910)_ = 17.19; *p* < 0.001; η_p_^2^ = 0.02	*F*_(4, 910)_ = 9.77; *p* < 0.001; η_p_^2^ = 0.04	Age × Country [*F*_(4, 910)_ = 2.96; *p* = 0.019; η_p_^2^ = 0.01], Time × Age [*F*_(1, 910)_ = 4.74; *p* = 0.03; η_p_^2^ = 0.01], Time × Country [*F*_(4, 910)_ = 27.71; *p* < 0.001; η_p_^2^ = 0.11] and Time × Age × Country [*F*_(4, 910)_ = 3.01; *p* = 0.017; η_p_^2^ = 0.01]
		Lebanon	17.5 ± 3	17.9 ± 3.4^*^				
		Italy	19.6 ± 3.6 L	20 ± 3.5^*^L				
		Spain	20.5 ± 4.1 L	19.9 ± 3.8				
		Portugal	17.3 ± 2.5IS	17.2 ± 2.5IS				
	11–14 years	Egypt	19.5 ± 3.9	19.2 ± 3.4^*^				
		Lebanon	19.8 ± 3.8Y	20.4 ± 3.7^*^Y				
		Italy	19.8 ± 4	20 ± 3.7				
		Spain	20.4 ± 3.7	20.3 ± 3.5				
		Portugal	18.5 ± 2.9S	18.8 ± 2.9				

^*^Significant difference compared to Pre; Y, significant difference compared to younger age group (8–10 years); E, significant difference compared to Egypt; L, significant difference compared to Lebanon; I, significant difference compared to Italy; S, significant difference compared to Spain; *p* < 0.05.

For BMI, the intervention program significantly reduced BMI only in Egypt for the 11–14 years age group (*p* < 0.05), while BMI increased significantly in Lebanon across both age groups and in Italy for the 8–10 years age group (*p* < 0.05). Significantly higher BMI values at the older age were observed only in the Lebanese population (*p* < 0.05). The highest BMI values were recorded in Italy and Spain for the 8–10 years age group, showing significantly higher values than Lebanon and Portugal (*p* < 0.05). At the older age group (11–14 years), no significant differences in BMI were observed between countries.

**Figure 1:**
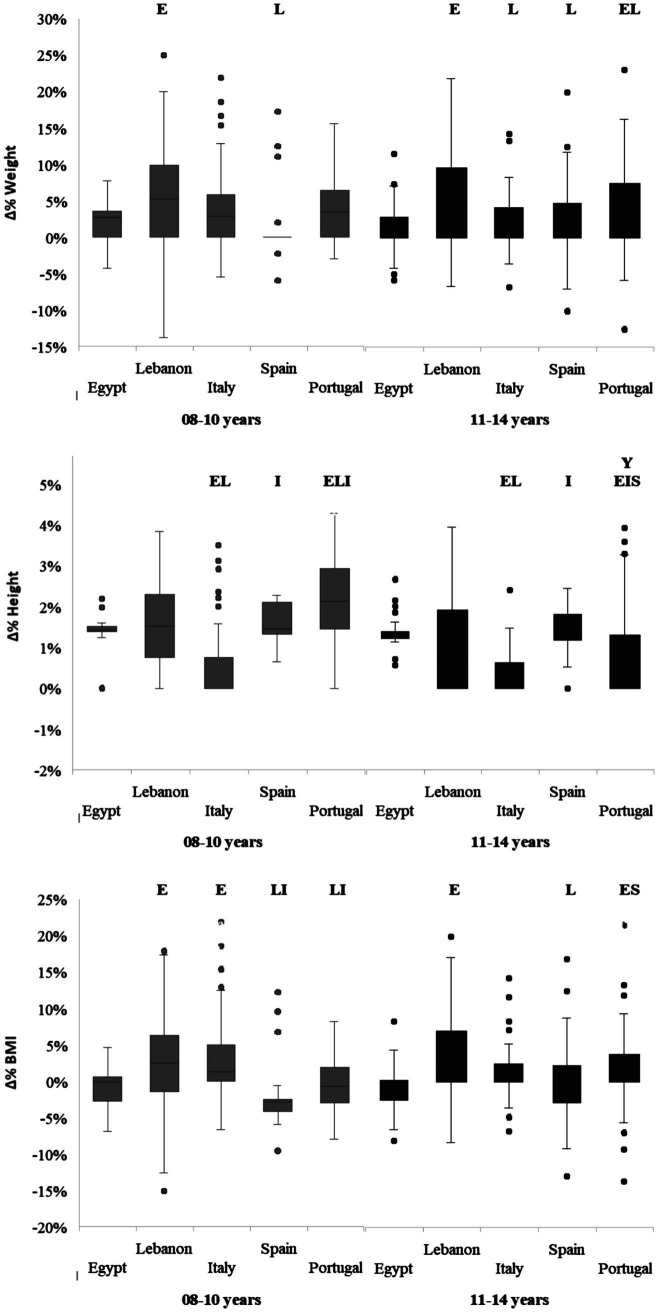
Percent change in body weight, Height, and BMI from pre- to post-intervention, categorized by age group and countries. Y, significant difference compared to younger age group (8–10 years); E, significant difference compared to Egypt; L, significant difference compared to Lebanon; I, significant difference compared to Italy; S, significant difference compared to Spain; at *p* < 0.05.

Figure 1 illustrates the effect of age and country on the percentage change in body weight, height, and BMI. The two-way ANOVA revealed significant Age × Country interactions for height [*F*(4,910) = 11.45, *p* < 0.001, ηp^2^ = 0.05] and BMI [*F* (4,910) = 2.66, *p* = 0.03, ηp^2^ = 0.01]. Significant main effects of age were found for height [*F*(1,910) = 63.73, *p* < 0.001, ηp^2^ = 0.07] and BMI [*F*(1,910) = 5.04, *p* = 0.02, ηp^2^ = 0.01], while significant main effects of country were observed for weight [*F*(4,910) = 19.04, *p* < 0.001, ηp^2^ = 0.08], height [*F*(4,910) = 40.85, *p* < 0.001, ηp^2^ = 0.15], and BMI [*F*(4,910) = 24.17, *p* < 0.001, ηp^2^ = 0.10]. Post-hoc analysis revealed that the highest increase in body weight was recorded in Lebanon for both age groups (5.9 and 5.1%, respectively), with significantly higher percentage changes compared to Egypt and Spain in both age groups, and to Portugal in the 11–14 years group (*p* < 0.05). For height percentage change, the highest increase was observed among Portuguese children aged 8–10 years (2.1%), which was significantly higher than in Egypt, Lebanon, and Italy (*p* < 0.05). For BMI percentage change, only Egypt and Spain showed negative changes in both the 8–10 years group (−1.5% and −2.5%, respectively) and the 11–14 years group (−1.4% and −0.5%, respectively), indicating a decrease in BMI. Post-hoc analysis revealed significant differences in BMI percentage change between Egypt and Lebanon in both age groups, Egypt and Italy in children, and Egypt and Portugal, as well as Spain and Portugal, in early adolescent (*p* < 0.05).

In terms of age effects, no significant differences were recorded between the two age groups in any country for both weight and BMI percentage changes. However, for height percentage change, only the Portuguese population showed a significant difference between age groups, with a higher change recorded in the 8–10 years group (*p* < 0.05).

### 3.2 Effects of the intervention program on physical fitness performances across different age groups and countries

Table 2 presents the effects of time, age, and country of living on physical fitness performance variables. The three-way repeated measures ANOVA revealed significant Time × Age × Country interactions for most variables (*p* < 0.001), except for JSW, which showed significant Time × Age and Time × Country interactions (*p* < 0.001). Significant main effects of time were observed for all variables (*p* < 0.001), and significant main effects of country were also recorded (*p* = 0.02 to *p* < 0.001). Significant main effects of age were found for most physical fitness performance variables (*p* = 0.03 to *p* < 0.001), except for the 6-min Run test.

**Table 2:** Physical fitness performance.

Variables	Age group	Country	Mean/SD		Main effect			Significant interaction
			Pre	Post	Time effect	Age effect	Country effect	
Sprint (sec)	8–10 years	Egypt	4.14 ± 0.49	4.19 ± 0.43	*F*_(1, 910)_ = 47.65; *p* < 0.001; η_p_^2^ = 0.05	*F*_(1, 910)_ = 11.81; *p* < 0.001; η_p_^2^ = 0.01	*F*_(4, 910)_ = 14.09; *p* < 0.001; η_p_^2^ = 0.06	Time × Age [*F*_(1, 910)_ = 86.93; *p* < 0.001; η_p_^2^ = 0.09] and Time × Age × Country [*F*_(4, 910)_ = 17.59; *p* < 0.001; η_p_^2^ = 0.07]
		Lebanon	4.12 ± 0.52	4.45 ± 0.44^*^				
		Italy	4.24 ± 0.57	4.09 ± 0.44 L				
		Spain	4.69 ± 0.53ELI	4.54 ± 0.53I				
		Portugal	4.13 ± 0.61S	4.33 ± 0.32				
	11–14 years	Egypt	4.32 ± 0.54	3.92 ± 0.4^*^				
		Lebanon	4.48 ± 0.53Y	3.87 ± 0.4^*^Y				
		Italy	4.28 ± 0.53	4.11 ± 0.33				
		Spain	4.41 ± 0.54	4.28 ± 0.53EL				
		Portugal	4.4 ± 0.52	3.83 ± 0.57^*^YS				
SLJ (cm)	8–10 years	Egypt	140.8 ± 27.9	148.8 ± 22.9	*F*_(1, 910)_ = 176.66; *p* < 0.001; η_p_^2^ = 0.16	*F*_(1, 910)_ = 7.51; *p* = 0.006; η_p_^2^ = 0.01	*F*_(4, 910)_ = 4.19; *p* = 0.002; η_p_^2^ = 0.02	Age × Country [*F*_(4, 910)_ = 4.81; *p* < 0.001; η_p_^2^ = 0.02], Time × Age [*F*_(1, 910)_ = 27.55; *p* < 0.001; η_p_^2^ = 0.03], Time × Country [*F*_(4, 910)_ = 9; *p* < 0.001; η_p_^2^ = 0.04] and Time × Age × Country [*F*_(4, 910)_ = 14.77; *p* < 0.001; η_p_^2^ = 0.06]
		Lebanon	140.4 ± 29.9	144.4 ± 21.7				
		Italy	136.1 ± 30.1	145.5 ± 20.4				
		Spain	121.6 ± 19.3	134.7 ± 22.5				
		Portugal	140.3 ± 34.9	148.8 ± 22.3				
	11–14 years	Egypt	137.6 ± 24.9	149.3 ± 20^*^				
		Lebanon	121.9 ± 26.8YE	155.5 ± 29.3^*^				
		Italy	142.1 ± 30.6 L	148.7 ± 19.3				
		Spain	142.3 ± 27.9YL	149.7 ± 26.3				
		Portugal	129.9 ± 24.5S	169.4 ± 26.3^*^YELIS				
JSW (n)	8–10 years	Egypt	29.2 ± 7.4	32.3 ± 5.6	*F*_(1, 910)_ = 555.66; *p* < 0.001; η_p_^2^ = 0.38	*F*_(1, 910)_ = 15.31; *p* < 0.001; η_p_^2^ = 0.02	*F*_(4, 910)_ = 10.52; *p* < 0.001; η_p_^2^ = 0.04	Time × Age [*F*_(1, 910)_ = 15.99; *p* < 0.001; η_p_^2^ = 0.02] and Time × Country [*F*_(4, 910)_ = 25.26; *p* < 0.001; η_p_^2^ = 0.1]
		Lebanon	30.3 ± 7.3	32.8 ± 6.5				
		Italy	29.4 ± 7.6	35.1 ± 5.5^*^				
		Spain	26.8 ± 8.5	32 ± 7.7^*^				
		Portugal	29.2 ± 7.4	37 ± 4.8^*^EL				
	11–14 years	Egypt	27.1 ± 6.7	31.4 ± 4.7^*^				
		Lebanon	25.2 ± 6.4Y	30.2 ± 5.3^*^				
		Italy	28.2 ± 7.4	34.5 ± 6.9^*^				
		Spain	24.9 ± 7.5	31.4 ± 7.5^*^				
		Portugal	26.1 ± 7	38 ± 6.6^*^ELS				
PU (*n*)	8–10 years	Egypt	14.6 ± 4.4	18.1 ± 4.2^*^	*F*_(1, 910)_ = 382.09; *p* < 0.001; η_p_^2^ = 0.3	*F*_(1, 910)_ = 4.57; *p* = 0.033; η_p_^2^ = 0	*F*_(4, 910)_ = 3.05; *p* = 0.017; η_p_^2^ = 0.01	Age × Country [*F*_(4, 910)_ = 2.47; *p* = 0.044; η_p_^2^ = 0.01], Time × Age [*F*(1, 910) = 5.59; *p* = 0.018; η_p_^2^ = 0.01] and Time × Age × Country [*F*(4, 910) = 4.89; *p* < 0.001; η_p_^2^ = 0.02]
		Lebanon	14.8 ± 5.2	18.8 ± 4.2^*^				
		Italy	14.8 ± 5.4	18.8 ± 4.9^*^				
		Spain	14.4 ± 4.4	19.1 ± 5.1^*^				
		Portugal	15.4 ± 4.8	17.7 ± 3.7				
	11–14 years	Egypt	14.6 ± 3.8	18 ± 3.3^*^				
		Lebanon	12.9 ± 3.9	19.2 ± 5.6^*^				
		Italy	15.8 ± 5.3	20.5 ± 5.1^*^				
		Spain	16.7 ± 6.4EL	19.8 ± 4.9^*^				
		Portugal	14.5 ± 4.8S	20.6 ± 4.9^*^YE				
SU (*n*)	8–10 years	Egypt	22.8 ± 6.7	25.3 ± 7.2	*F*_(1, 910)_ = 214.1; *p* < 0.001; η_p_^2^ = 0.19	*F*_(1, 910)_ = 20.29; *p* < 0.001; η_p_^2^ = 0.02	*F*_(4, 910)_ = 5.61; *p* < 0.001; η_p_^2^ = 0.02	Age × Country [*F*_(4, 910)_ = 9.52; *p* < 0.001; η_p_^2^ = 0.04], Time × Age [*F*_(1, 910)_ = 41.74; *p* < 0.001; η_p_^2^ = 0.04], Time × Country [*F*_(4, 910)_ = 3.05; *p* = 0.016; η_p_^2^ = 0.01] and Time × Age × Country [*F*_(4, 910)_ = 15.35; *p* < 0.001; η_p_^2^ = 0.06]
		Lebanon	21.5 ± 6.8	21.7 ± 5.4				
		Italy	22.4 ± 7.6	27.3 ± 5.4^*^L				
		Spain	19.6 ± 6.2	22.9 ± 6.9				
		Portugal	22.2 ± 6.8	23.1 ± 7.4				
	11–14 years	Egypt	21 ± 6.5	25.5 ± 5.8^*^				
		Lebanon	18.2 ± 6.8	27.3 ± 5.6^*^Y				
		Italy	22.1 ± 7.9	27.3 ± 6.2^*^				
		Spain	29.2 ± 11.8YELI	30.9 ± 11.5YEL				
		Portugal	20 ± 6.6S	29.8 ± 7.6^*^YE				
Run (m)	8–10 years	Egypt	939 ± 165	1,161 ± 161^*^	*F*_(1, 910)_ = 1546.87; *p* < 0.001; η_p_^2^ = 0.63	*F*_(1, 910)_ = 0.04; *p* = 0.838; η_p_^2^ = 0	*F*_(4, 910)_ = 5.37; *p* < 0.001; η_p_^2^ = 0.02	Age × Country [*F*_(4, 910)_ = 3.22; *p* = 0.012; η_p_^2^ = 0.01], Time × Age [*F*_(1, 910)_ = 12.09; *p* < 0.001; η_p_^2^ = 0.01] and Time × Country [*F*_(4, 910)_ = 5.06; *p* < 0.001; η_p_^2^ = 0.02]
		Lebanon	944 ± 190	1,184 ± 158^*^				
		Italy	952 ± 186	1,187 ± 150^*^				
		Spain	854 ± 157	1,090 ± 136^*^				
		Portugal	938 ± 196	1,219 ± 131^*^				
	11–14 years	Egypt	890 ± 165	1,161 ± 152^*^				
		Lebanon	856 ± 150Y	1,179 ± 125^*^				
		Italy	972 ± 204 L	1,226 ± 154^*^				
		Spain	908 ± 202	1,156 ± 157^*^				
		Portugal	872 ± 188	1,226 ± 199^*^				

^*^Significant difference compared to Pre; Y, significant difference compared to younger age group (8–10 years); E, significant difference compared to Egypt; L, significant difference compared to Lebanon; I, significant difference compared to Italy; S, significant difference compared to Spain; *p* < 0.05.

Sprint performance significantly improved (decrease in time) after the intervention in most countries, except for Italy and Spain in the 11–14 years age group (*p* < 0.05), while no significant improvement was observed in any country for the 8–10 years age group. Better performance in the older age group was recorded only in Lebanon and Portugal (*p* < 0.05). At the younger age (8–10 years), Italian children outperformed Spanish participants, who also showed the worst performance in the older age group compared to those from Egypt, Lebanon, and Portugal.

SLJ performance improved significantly only in the 11–14 years age group, specifically in Egypt, Lebanon, and Portugal (*p* < 0.05). Overall, SLJ performance declined with older age, particularly in the Lebanese, Spanish, and Portuguese populations. Performance was comparable across countries in the younger age group (8–10 years), while Portugal showed significantly better performance post-intervention in the older age group (11–14 years; *p* < 0.05).

JSW performance improved significantly from pre- to post-intervention in most countries and age groups (*p* < 0.05), except in Egypt and Lebanon for the younger age group. The highest post-intervention performance was recorded in the Portuguese population across both age groups (*p* < 0.05), with generally comparable values between age groups in all countries.

PU performance improved significantly from pre- to post-intervention in most countries and age groups (*p* < 0.05), except in Portugal for the younger age group. Performance was generally comparable between age groups and countries, except for Portugal, which showed higher performance in the older age group and outperformed Egypt at this age (*p* < 0.05).

SU performance improved significantly only in Italy for the 8–10 years age group and in most countries for the 11–14 years age group (*p* < 0.05), except in Spain. Older age groups showed higher performance in Lebanon, Spain, and Portugal (*p* < 0.05). Post-intervention performance was higher in Italy compared to Lebanon for the younger age group and higher in Spain and Portugal for the older group (*p* < 0.05).

Endurance performance during the 6-min running test improved significantly in both age groups across all countries (*p* < 0.05), with generally comparable results between age groups and countries.

**Figure 2:**
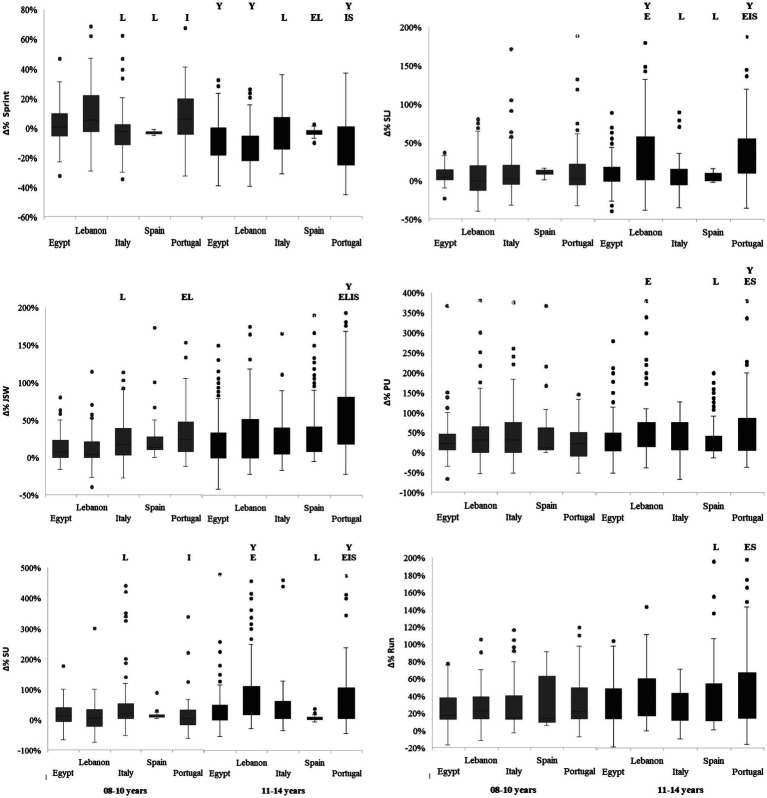
Percent change in physical fitness variables from pre- to post-intervention, categorized by age group and countries. Y, significant difference compared to younger age group (8–10 years); E, significant difference compared to Egypt; L, significant difference compared to Lebanon; I, significant difference compared to Italy; S, significant difference compared to Spain at *p* < 0.05.

Figure 2 illustrates the effects of age and country on the percentage change in various physical fitness performance variables. The two-way ANOVA revealed significant Age × Country interactions for sprint, SLJ, PU, and SU, with *p*-values ranging from 0.003 to <0.001 and ηp^2^ values between 0.02 and 0.08. Significant main effects of age were found for most variables, except PU, with *p* < 0.001 and ηp^2^ values between 0.01 and 0.09. Significant main effects of country were observed for SLJ, JSW, SU, and the 6-min Run, with p-values ranging from 0.002 to <0.001 and ηp^2^ values between 0.02 and 0.06.

Post-hoc analysis revealed that, for most studied parameters, the most significant differences between countries were observed in the 11–14 years age group. For sprint percentage change, the greatest improvements (largest decreases) were recorded in Lebanon (−13.7%) and Portugal (−12.9%), followed by Egypt (−9.3%) in the 11–14 years age group, with significant differences compared to Spain and Italy (*p* < 0.05). In contrast, improvements in the 8–10 years age group were minimal, not exceeding 3.4% (as observed in Italy and Spain), while the remaining countries showed worsened performance (positive percentage changes). For SLJ percentage change, the highest improvements were observed in Lebanon and Portugal (28–30%) in the 11–14 years age group, which were significantly higher than those in Spain and Italy (*p* < 0.05). However, in the younger age group (8–10 years), percentage changes were comparable across countries (*p* > 0.05).

JSW performance showed positive percentage changes across all countries in both age groups. In the 8–10 years age group, the highest improvement was recorded in Portugal (27%), followed by Italy and Spain (19%). In the 11–14 years age group, Portugal again exhibited the highest improvement (46%), which was significantly greater than the percentage changes observed in the other countries (*p* < 0.05).

PU performance followed a similar positive trend. In the 8–10 years age group, the highest improvement was observed in Spain (32.5%), followed by Lebanon and Italy (27%), with no significant differences between countries. In the 11–14 years age group, the highest improvements were recorded in Lebanon (49%) and Portugal (43%), which were significantly higher than those in Spain and Egypt (*p* < 0.05).

Positive percentage changes in SU performance were observed across all age groups and countries. In the 8–10 years age group, the highest improvement was recorded in Italy (22%), showing significant differences compared to Lebanon and Portugal (*p* < 0.05). In the 11–14 years age group, the greatest improvements were observed in Lebanon and Portugal (approximately 50%), which were significantly higher than those in Egypt, Italy, and Spain (*p* < 0.05).

Endurance performance registered positive percentage changes across all age groups and countries, with comparable improvements ranging from 24 to 30% in the 8–10 years age group (*p* > 0.05). In the 11–14 years age group, the highest values were recorded in Portugal (40.5%) and Lebanon (38%), which were significantly higher than those in Spain (*p* < 0.05) and Egypt (when compared to Portugal, *p* < 0.05).

In terms of age effects, significantly higher improvements at the 11–14 years age group compared to 8–10 years were primarily observed in Portugal across all physical fitness variables except Run (*p* < 0.05), in Lebanon for Sprint, SLJ, and SU (*p* < 0.05), and in Egypt for Sprint only (*p* < 0.05).

## 4 Discussion

### 4.1 Main findings and interpretations

Our findings demonstrate that the school-based PA intervention led to significant improvements in several components of health-related fitness—specifically speed, lower-body power, coordination, muscular endurance, and cardiovascular endurance—across all five Mediterranean countries. However, these improvements were not uniform. Early adolescents (11–14 years) generally experienced greater gains than younger children (8–10 years), and the magnitude of improvement varied between countries. For instance, early adolescents in Lebanon and Portugal exhibited notable improvements in sprint performance and endurance capacity, while younger children in those and other countries showed more modest changes. Some groups, particularly the younger age group, did not significantly improve in certain tests such as the 20 m sprint. This suggests that the intervention’s effects were moderated by developmental stage. This age-related disparity aligns with physiological evidence that prepubertal children typically show a reduced training response, achieving only about half the gains seen in postpubertal individuals under similar exercise conditions (48, 49). Therefore, the greater gains among early adolescents in our study may reflect their higher responsiveness to training, whereas younger children may benefit from longer or more intensive interventions. Additionally, the pronounced improvements observed in speed and strength are likely driven by rapid neuromuscular adaptations, such as enhanced motor unit recruitment, firing frequency, and coordination efficiency (50). In contrast, improvements in endurance capacity require more time, as they depend on adaptations of the cardiovascular and respiratory systems—including increased stroke volume, enhanced capillary density, and improved mitochondrial function—which involve more extensive tissue remodeling and slower physiological processes (51, 52). This difference in adaptation timelines helps explain why gains in speed and strength were more immediate, whereas endurance improvements were more gradual.

Cross-country differences in outcomes were also evident and align closely with our data, underscoring the influence of contextual factors on intervention effectiveness. For instance, Portugal and Lebanon not only had high baseline potential for improvement but also realized the greatest post-intervention gains in several fitness components (such as push-ups, sit-ups, and endurance run), whereas Spain and Italy showed smaller improvements in some measures. Countries with lower baseline fitness (e.g., Lebanon, Portugal) showed the greatest improvements, while fitter countries (e.g., Spain) exhibited smaller gains, possibly due to a ceiling effect. By the end of the intervention, previously observed fitness disparities between countries were somewhat altered: for example, Portuguese early adolescents showed the greatest improvements in endurance, followed by Lebanese and Spanish peers, with all three outperforming the younger groups. Our findings confirm that universal school-based programs may not yield uniform results, as differences in curricula, sports access, and initial fitness influence outcomes (36, 53). Factors such as variations in physical education curricula, extracurricular sports opportunities, and baseline fitness likely contributed to the differing country-specific responses (3). Moreover, the effectiveness of these interventions is significantly influenced by the implementation setting and fidelity. Elements such as school leadership, policies, workforce structure, program characteristics, and the overall school environment play critical roles in shaping outcomes (54). High fidelity and quality implementation, along with positive adaptations, have been shown to enhance intervention impact. For instance, schools that implemented interventions with high fidelity and quality demonstrated more favorable results (55). As prior cross-cultural research suggests, disparities in how children engage in organized PA (due to school systems or community sports) can influence fitness outcomes (36). Our results reinforce that notion, implying that culturally and contextually tailored approaches might be necessary to maximize the effectiveness of school-based fitness programs in each setting.

Importantly, the intervention’s effect on anthropometric measures was minimal. Weight and height increased with growth, but stable BMI indicates healthy, proportionate development. In other words, improved fitness did not come at the cost of unhealthy weight changes. This outcome is encouraging, as it suggests that participants’ weight gain was likely attributable to normal growth and possibly increased muscle mass rather than excess fat. Maintaining a stable BMI while improving fitness levels is a positive indicator that the program supported healthy development. It also aligns with the understanding that short-term interventions in children often do not drastically change BMI, especially when concurrent growth in stature is occurring. Our findings here mirror those of the Egyptian pilot study, which found no significant change in BMI alongside improvements in certain fitness components, underscoring that fitness gains can be achieved without immediate changes in body composition (29). This observation is consistent with prior research showing that while PA interventions can significantly enhance aerobic and anaerobic fitness, they often do not result in substantial changes in anthropometric indicators, particularly over shorter durations (56). This has important public health implications: a well-designed physical activity program can enhance functional fitness and health markers in children even if weight metrics remain constant in the short term.

### 4.2 Comparison with previous studies

The outcomes of our study align with previous research on school-based PA interventions and extend the literature by incorporating a multi-country, cross-cultural perspective. Numerous studies have shown that increased PA in school settings leads to improvements in children’s aerobic and muscular fitness (29–32). For example, interventions that increased the frequency of physical education classes reported significant gains in aerobic capacity and muscular strength (34). Our findings corroborate these results, with observed improvements in endurance (6-min run) and strength-based measures (standing long jump and push-ups) across many groups. That these gains were achieved through a relatively short-term program highlights children’s responsiveness to increased PA, consistent with findings from the Fit-4-Fun trial and similar school-based interventions (57). Furthermore, the intervention’s effectiveness across diverse populations supports the broader applicability of structured programs and reinforces calls for their wider implementation.

Beyond general intervention effects, our study contributes novel insights into how cultural and environmental factors might modulate outcomes. Prior work by Aly et al. (36) documented significant baseline differences in fitness levels among children from Egypt, Italy, Lebanon, Portugal, and Spain, attributing these disparities to variations in PA opportunities and lifestyles across countries (36). The current intervention study builds on those findings by showing that when a standardized PA program is applied, the relative improvements are inversely related to some of those baseline rankings. This suggests that children from countries with lower initial fitness (potentially due to fewer routine PA opportunities) can “catch up” to some degree when given equal access to structured exercise. For instance, Lebanese children—who had certain fitness disadvantages at baseline relative to peers from Spain—achieved among the highest gains in measures like sprint speed and muscular endurance during the intervention. Conversely, Spanish children, who on average started with superior performance in several tests, showed smaller proportional improvements. This inverse relationship between baseline fitness and improvement magnitude has been observed in other contexts and likely reflects a regression to the mean or greater room for improvement among initially less-fit groups (58). It also emphasizes the value of providing quality PA programs in regions where children’s habitual fitness may be lagging; these children stand to benefit substantially from interventions.

Our age-related findings are consistent with, and build upon, previous research showing that older children and adolescents typically experience greater gains in strength and aerobic capacity than prepubertal children, largely due to hormonal and developmental differences (59). The pronounced improvements in the early adolescence group—particularly in strength-oriented tests like push-ups and sit-ups in countries such as Lebanon and Portugal—support this trend. In contrast, the more modest gains among 8–10 year-olds mirror findings from short-term interventions where younger children demonstrate improvements, though less pronounced (48, 49). Nonetheless, the younger cohort in our study did achieve meaningful gains, especially in coordination and endurance, across several countries. This suggests that with well-designed programs, prepubescent children can still benefit from PA, albeit to a lesser extent. These insights are valuable for educators and practitioners, highlighting the potential of tailored, higher-intensity or play-based interventions to better engage younger children, as recommended by pediatric exercise guidelines.

In comparing specific fitness components, our multi-country results partly contrast with the single-country pilot study conducted in Egypt under the DELICIOUS project. That 6-week pilot intervention reported significant improvements primarily in coordination (jumping sideways) and lower-body power (standing long jump), with no significant changes in sprint speed, upper-body strength, or abdominal strength observed (29). In the present study, which spanned a longer duration and included a larger, more diverse sample, we observed significant improvements in nearly all those fitness domains (including sprint speed and muscle strength/endurance) in at least some subgroups, particularly among older children. This difference suggests that a longer or more comprehensive program can elicit broader fitness enhancements than a brief pilot, and that factors such as participant age and baseline fitness play a critical role in determining which fitness components improve. It’s worth noting that aerobic endurance improved markedly in our study across all countries (with 6-min run performance increasing ~24%–40% depending on group), whereas the pilot showed only marginal gains in endurance (perhaps due to its shorter timeframe). Thus, our findings provide stronger evidence that the “Be Fit” style school-based program can improve cardiovascular fitness when implemented at scale, while also confirming the pilot study’s implication that certain capacities (speed, strength) may require more time or intensity to change significantly. Overall, the consistency of our core outcomes with existing literature lends credibility to the effectiveness of school-based interventions, and the discrepancies (such as those with the pilot study) offer learning opportunities on how to optimize these programs.

### 4.3 Limitations and future directions

While this study offers valuable insights, several limitations must be acknowledged to ensure accurate interpretation of the findings. First, the absence of a control group in our design limits the ability to attribute improvements solely to the intervention, since natural growth and maturation over the study period could also enhance fitness measures. To address this limitation, we focused on age-specific changes within the broader context of cross-country comparisons. Notably, early adolescents—those approaching or undergoing puberty—demonstrated disproportionately greater improvements than younger peers, with the magnitude of change varying by country. This pattern cannot be fully attributed to natural growth alone, suggesting potential effects of both the training intervention and cultural context. While these findings provide meaningful insight, a future controlled trial would be necessary to establish stronger causal inferences. Second, there were inherent differences in sample characteristics between countries (e.g., baseline fitness levels, cultural attitudes toward PA, and perhaps slight variations in program implementation) that could have influenced the outcomes. We treated the intervention as a uniform exposure, but adherence and enthusiasm might have varied by site, introducing some bias. Although we believe that analyzing percentage change helps minimize the impact of baseline heterogeneity, future studies should assess program fidelity and possibly adjust for baseline fitness or use each country as its own control to better understand these context effects. Third, biological maturation (e.g., proximity to peak growth velocity) was not assessed. Future interventions should consider including maturity status estimations to better interpret changes in physical fitness and anthropometric outcomes across different developmental stages. A further limitation is that the intervention may have been insufficient for some fitness components, especially among younger children, to show full adaptation. Longer interventions or follow-up assessments would help determine whether gains are sustained or continue to increase. We also did not examine sex-based differences, which is a limitation given the developmental differences between boys and girls in this age group. Future work should explore gender-specific responses to the intervention in diverse culture contexts. Importantly, while this study focused on PA, other influential factors—such as nutrition and adherence to the Mediterranean diet (a focus of the broader DELICIOUS project)—were not analyzed. These factors likely interact with physical fitness outcomes and should be considered in future research. Finally, these findings highlight the need for supportive environments that enable children and adolescents to engage in regular PA. Beyond school-based interventions, broader policy initiatives are essential to promote sustainable PA habits. Policymakers should consider integrating PA into national education agendas, ensuring supportive school infrastructure, and prioritizing active lifestyles in youth development strategies. Creating such environmental and policy conditions is critical to addressing the inactivity crisis and promoting long-term public health. Another important consideration involves data privacy and ethical data sharing practices. Given the sensitive nature of collecting fitness and health data from children across culturally diverse settings, future research should incorporate rigorous safeguards for participant confidentiality. Recent methodological advancements, such as those described by Zhang et al. (60) offer useful frameworks for balancing open science practices with ethical data stewardship. Adopting such frameworks is essential to ensure that cross-national studies involving minors remain compliant with evolving standards of transparency and privacy protection.

Despite these limitations, the study’s strengths include its large, diverse sample and the cross-cultural comparative approach, which is uncommon in school-based fitness intervention research. By encompassing five countries, we could discern patterns that a single-country study might overlook, thereby providing a more holistic understanding of how context interacts with a PA program’s effectiveness.

## 5 Implications and conclusions

In summary, this study provides robust evidence that a structured, school-based PA program can significantly enhance children’s fitness across different domains (speed, strength, endurance, and coordination) in a variety of Mediterranean settings. The improvements observed, particularly among early adolescents, have meaningful implications for public health and education—healthier fitness profiles in youth are associated with numerous immediate and long-term benefits, including better cardiovascular health, muscular development, and even academic performance (1, 3, 7, 61–63). Our cross-country findings emphasize that while the foundational benefits of exercise are universal, programs may need to be tailored to account for age and cultural context to maximize their impact. Schools in regions where baseline fitness is low might expect especially large benefits from implementing such interventions, whereas those in settings with more active youth might focus on maintaining engagement and reaching the less-fit subgroup of students. The fact that BMI remained stable suggests that fitness improvements were achieved alongside healthy growth, without adverse changes in body composition.

Going forward, educators and policymakers should consider integrating regular PA sessions into school curricula as a strategy to improve youth fitness and combat sedentary lifestyle trends. Our results support recommendations for culturally adapted physical education strategies (36)—for instance, incorporating traditional games or locally favored activities to increase participation—and for paying special attention to motivating younger children who may not respond as readily. Furthermore, establishing international collaborations (like the DELICIOUS project) can facilitate knowledge exchange on effective practices and help develop guidelines that recognize both universal principles of child fitness and local needs. Ultimately, our findings affirm that investing in school-based PA programs is a viable and effective approach to enhance physical fitness among children, and they highlight the importance of refining these programs to ensure that all children, regardless of age or country, can achieve optimal benefits.

## Data Availability

The raw data supporting the conclusions of this article will be made available by the authors, without undue reservation.
